# Functional Genomics Thickens the Biological Plot

**DOI:** 10.1371/journal.pbio.0030219

**Published:** 2005-06-14

**Authors:** Virginia Gewin

## Abstract

New functional genomic tools are enabling researchers to draw on a large cast of 'non-model' organisms to help set the stage for ecological and evolutionary discovery.

Just a handful of species are the basis for a staggering amount of our biological knowledge. From the ever-popular mouse *(* Mus musculus *)* to the always fruitful fruit fly *(* Drosophila melanogaster *)*, biologists have cultivated a cadre of model organisms to unravel the intricate mysteries of cell communication, genetics, and embryonic development. “Model system,” however, is a tricky term to define. The biological superstars are seven genetic organisms—yeast *(* Saccharomyces cerevisiae *)*, Escherichia coli, fruit fly, roundworm *(* Caenorhabditis elegans *)*, mustard plant *(* Arabidopsis thaliana *)*, zebrafish *(* Danio rerio *)*, and mouse—for which “model organism” has become shorthand. Although a second tier of organisms can be cast as models when they facilitate study of a particular biological process, they are often merely supporting actors relegated to a bit part on the biological stage.

For all the laboratory tales these seven model species have helped to tell, there remains a wealth of evolutionary and ecological questions still to be addressed. Understanding organisms' responses to mutations and the environment in order to paint a more complete story of biological networks is the biggest challenge in biology today [1]. In addition to providing insight into ecology and evolutionary lineages, studies of nonmodel organisms are sure to reveal as-yet unknown biological mechanisms.

Recently, the plot thickened when genomic data revealed the amazing degree to which genes are conserved throughout species. Surprisingly, it's not simply the genes, but their regulation, that gives rise to the remarkable diversity of creatures. New molecular techniques take advantage of these findings to reveal gene expression and function in an expanded cast of characters. Functional genomics makes nonmodel organism studies more robust—blurring the line between model and nonmodel species and setting the stage for synergistic discoveries in evolutionary biology and ecology.

## A Star Is Born

Previously held assumptions are already being reconsidered in the face of growing amounts of genomic data. The sea anemone may seem an unlikely character to shake the phylogenic tree, but new genomic evidence suggests that the sea anemone is more closely related to the greater majority of more complex animals than its position above the lowly sponge had once presumed [2]. “Our ideas of evolution—who gave rise to what—are changing rapidly as we get more data from more animals,” says Linda Holland, evolutionary biologist at University of California at San Diego.

**Figure 1:**
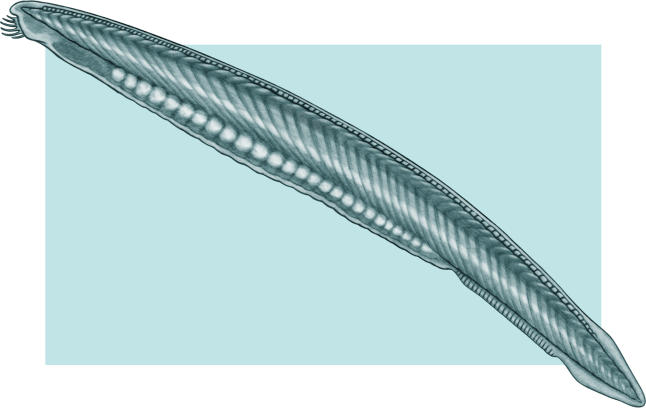
Amphioxus, a Small, Translucent, Fish-Like Animal That Is the Closest Living Invertebrate Relative of the Vertebrates Its strategic position on the phylogenetic tree is enough to provide valuable insights—worthy of the years of work to perfect techniques already standard in the superstar model species. (Image: Giovanni Maki)

Holland is one of the few researchers to study amphioxus, a small, translucent, fish-like animal that is the closest living invertebrate relative of the vertebrates (Figure 1). She likens the use of such simple species to studying architecture. “You want to determine the architecture of the simple church before examining the gargoyles that vertebrates erect on the system,” she says. Its strategic position on the phylogenetic tree is enough to provide valuable insights—worthy of the years of work to perfect techniques already standard in the superstar model species. “Its genome is as close as you can get to the ancestral vertebrate genome,” she says.

Amphioxus is a perfect example of grooming a starlet model organism using today's techniques. Holland started out cloning genes and looking for gene expression. Determining when a gene is expressed is a first step toward understanding its function. When she and colleagues documented expression of an amphioxus counterpart (a “homolog”) of a crucial vertebrate development gene, called *Hox3*, they began to assemble genomic tools to exploit this organism's potential as a model for vertebrate development.

Although only a few organisms have a sequenced genome to add to their reagent list, Douglas Crawford, evolutionary biologist at the University of Miami, argues that genomic tools, such as a cDNA library—cloned complementary DNA molecules synthesized from expressed RNA—are affordable enough to be accessible to any research group that has the considerable time it takes to sequence and, more importantly, annotate genes. With the library comes the ability to create microarrays with which to study gene expression.

Microarrays are sets of DNA sequences affixed to glass slides that enable researchers to determine which of the thousands of genes in an organ, organism, or population are expressed at any given time. The microarray is a revolutionary tool to cast widely for clues to gene function. “Thus far, gene functions could be studied only with the help of those model systems allowing genetic analysis,” says Daniel Chourrout, director of the Sars International Centre for Marine Molecular Biology, University of Bergen, Norway.

However, many interesting behavioral, physiological or ecological traits and responses are poorly expressed or absent in the genetic model organisms. Those behavioral genes that are known to model species instantly become candidate genes to search for in nonmodel organisms. Comparative genomics has changed the playing field for understanding mechanisms and evolution of behavior—genes being the common currency. For example, once the social honeybee's genome sequence is complete, its gene-behavior relationships can be compared to the solitary fruit fly.

“The biggest problem with model organisms is that they are inbred strains,” Crawford says. Restricting an evolutionary view to lab strains could miss important signals, such as epistasis, control of a phenotype by two or more genes. The microarray enables researchers to probe those organisms most suited to answer a specific biological question [3]. Crawford developed microarrays to assess the fitness of Fundulus heteroclitus, a fish species that lives along a steep thermal gradient in the Atlantic Ocean—from the cold north to the warmer south. Highlighting the functional importance of DNA polymorphisms, he has been able to show a biologically relevant difference in the expression of metabolic genes among individuals in a population.

Other organisms are similarly poised to answer ecological questions. Researchers studying the freshwater crustacean *Daphnia (* Daphnia pulex *)*—a toxicologically sensitive organism that plays a key role in freshwater ecology—are undertaking similar strategies to become ingénue genetic and genomic systems (Box 1).

**Box 1. Today's Model Toolkit**

**Figure 2:**
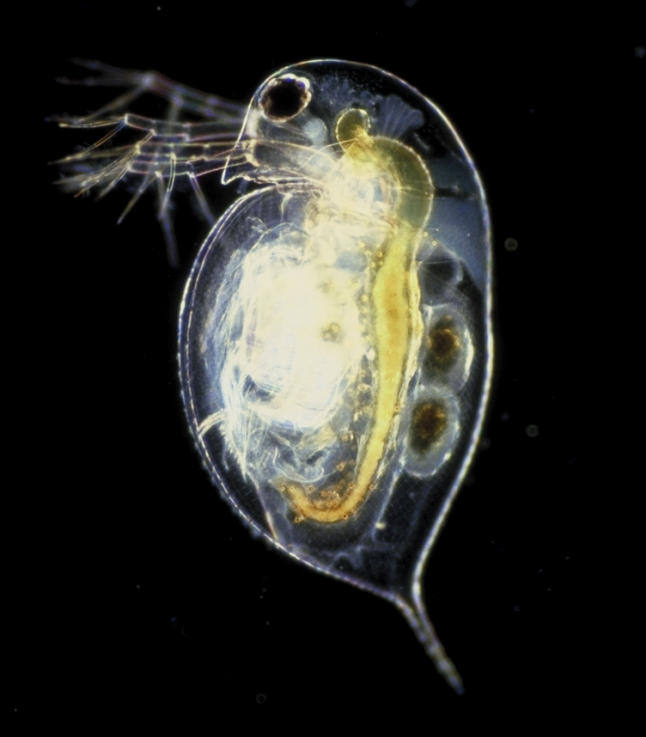
Daphnia pulex, a Species Waiting in the Wings to Achieve “Model” Status (Photo: Paul Hebert)

Using *Daphnia* (Figure 2)—a species showing adaptive traits that keep re-emerging to cope with a variety of ecological conditions—researchers want to detail what ecology can tell us about genomics.

**Figure 3:**
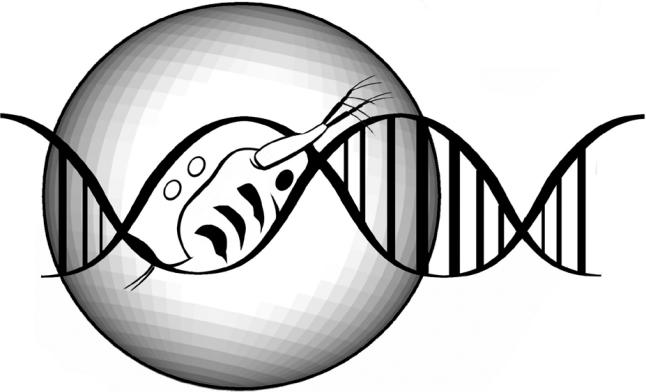
The *Daphnia* Genomics Consortium Logo (Design: S. Lourido)

Indeed, the Daphnia community is actively assembling the elements to achieve status as a model genetic system. “We're trying to not necessarily build tools for what is considered at this point a nonmodel—we're trying to create a model system,” says John Colbourne, founding member of the *Daphnia* Genomics Consortium (Figure 3). To do so, they must assemble the genetic tools that are the defining feature of traditional model genetic species as well as functional genomics approaches. The first act was to create a consortium of the relevant researchers. Those researchers are now assembling cDNA libraries to make microarrays, finding ways to transform the organism using knockout techniques, developing cell lines assembling genetic and physical maps, and creating a customized electronic database. To complete the kit—the genome is on its way.

Such a toolkit for a “model” nonmodel species is getting easier to assemble, particularly given the ease of creating a cDNA library. But until a genome is in hand, the easiest way to achieve status can always benefit from a robust classical biological history, a vocal or influential research community, or a critically understudied position on the phylogenetic tree.

## Phenotype Casting

Until systems such as *Daphnia* take hold, there are two ways to get the most information from ecologically relevant populations—take the model genetic system to a natural setting or apply new techniques to nonmodels [4]. While some inventive researchers are finding interesting results with the former method (Box 2), ecologists are increasingly interested in the genes that underlie native organism fitness in order to shed light on environmental modifications to gene expression [5].

**Box 2. Reality Ecology**

**Figure 4:**
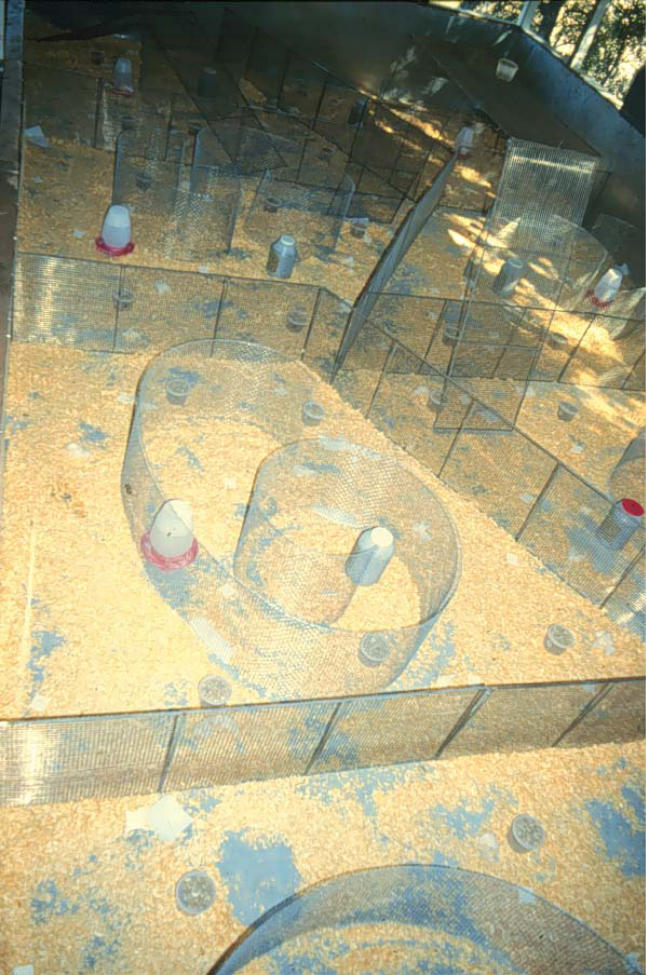
Setting the Stage in Ecology—The Phenotron (Image: Wayne Potts)

A typical ecologist is not usually inclined to study mice. But Wayne Potts is not typical. He has designed a phenotron (Figure 4), a man-made enclosure replete with three-dimensional complexities, otherwise known as a mouse barn. Using wild mice, he studies the effects of stressful situations found in the real-world social ecology of these animals. The findings are startling. He's been able to show that the impacts of inbreeding are far greater than previous studies detected using lab assays. Taking offspring from one generation of full sib inbreeding and then allowing them to compete against outbred controls in the phenotron, Potts found an additional five-fold reduction in male fitness. “If you mate with your sister, your sons are effectively dead,” he says. Extrapolating from this finding, he cautions against assuming that gene knockouts with little or no phenotypic effect means there is negligible impact on the organism. “If you care about gene function, you've got to test them under the competitive conditions in which the genes evolved,” he says.

“Ecology is about phenotypes, and our goal is to understand the phenotypic variation that matters in a natural context,” says Thomas Mitchell-Olds, plant ecologist at the Max Planck Institute for Chemical Ecology in Jena, Germany. Mitchell-Olds combines both approaches—sticking to wild relatives of the best genetic plant model Arabidopsis in order to take advantage of the already established experimental methods that allow him to focus on hypotheses in undisturbed environments. Studying wild relatives has one additional advantage: candidate genes responsible for ecological variation, such as resistance to insects and pathogens, drought tolerance, and flowering, have already been identified in Arabidopsis. Mitchell-Olds can clone these genes in the wild relatives he studies to see if they have the same function.

In addition to exploring natural phenomena, ecologists are using microarrays to determine the gene-expression changes related to exposure to existing and emerging contaminants, including pharmaceutical compounds, pesticides, and nitrogen inputs from agriculture—a “canary on a chip” capable of assessing environmental impacts on an organism's reproduction and fitness [6]. “These techniques provide information about genetic mechanisms pertaining to physiology and behavior of organisms and how environment influences phenotype, either as a result of natural variables or toxicology,” says Rebecca Klaper, ecologist at the Great Lakes WATER Institute, at the University of Wisconsin-Milwaukee.

Klaper studies the lake sturgeon *(* Acipenser fulvescens *)*, a complex yet tragic character that has an estimated 250 chromosomes, some of which are very small. For long-lived, threatened, or endangered species, such as the lake sturgeon, comparing cDNA libraries from different tissues and time points to known databases allows them to identify differentially regulated genes depending on reproductive stage or exposure to toxins. Ultimately, they will combine these techniques with home-grown microarrays for this species that could never be raised in the lab. They hope to better understand how evolutionarily ancient sturgeon are affected by toxins, how their immune system functions, how sexual development and reproductive stage are determined, and what mechanisms are responsible for cueing this development.

## Evo-Devo-Tees

Linking phenotype to genotype, or—broadly—form to function is the core of evolutionary biology. Gene function is crucial to understand how evolution developed new body forms. “To really understand the evolution of development, you have to sample pretty broadly,” says Nipam Patel, evolutionary biologist at the University of California at Berkeley. “Between mice and flies, you see conservation of genes, but that doesn't tell how evolution changes body plans and morphologies,” he adds.

“Expression studies tell you about conservation of expression but nothing on the conservation of function,” says Gregor Bucher, developmental biologist at Göttingen University in Germany. Indeed, verification of function is the key step, often accomplished via transgenesis—incorporating an introduced gene that can be transmitted to successive generations—which is not an option for most species.

In lieu of transgenesis, evo-devo-tees increasingly favor reverse genetics—knocking out a specific known gene to look for a change in phenotype—as opposed to the more robust, traditional method of forward genetics, which relied on induced mutations. Two techniques, RNA interference (RNAi) and oligonucleotide morpholinos, have been used successfully to effectively knock out specific genes in nonmodel systems. RNAi degrades RNA, causing reduced expression, while morpholinos block translation of proteins. In addition to being ridiculously easy to deliver in some species, RNAi has one added benefit: injecting pregnant mothers of some species creates knock-down embryos. Indeed, knock-down embryos work well for the red flour beetle *(* Tribolium castaneum *)*, which is a more representative species than the fruit fly to research arthropod head development and segmentation. The fruit fly's head forms all at once instead of in the anterior-to-posterior progression usual to most arthropods. Like many other evo-devo researchers, Thomas Kaufman, evolutionary biologist at Indiana University and fruit fly devotee, is now exploring nonmodel species such as the milkweed bug *(* Oncopeltus fasciatus *)*. He used RNAi to show that genes controlling mandible mouthparts in the fruit fly produce specialized piercing-sucking mouthparts in the milkweed bug. Such seemingly subtle differences represent regulatory paradigms differentiating evolution between orders of insects.

Unfortunately, RNAi doesn't work in every species, or even every gene. And “one has to be careful about interpretations of phenotype,” Crawford says. Often, oligonucleotide morpholinos can serve as a stand-in for the popular RNAi.

Once Holland exhausted the utility of gene-expression patterns to infer homologies of structures in amphioxus, her research group moved from gene-expression patterns to mechanistic studies using oligonucleotide morpholinos to knock down gene function. It took about five years to work out the techniques, particularly since amphioxus eggs are currently available only about 15 nights out of the year. But the amphioxus genome has only single copies of most development genes, providing a straightforward route to interpret functional knock-downs. Using morpholinos, they are starting to put together an account of developmental patterning that may serve as a model for vertebrate systems.

For all the excitement surrounding these new techniques, good old-fashioned forward genetics has allowed the three-spined stickleback *(* Gasterosteus aculeatus *)* to quickly achieve supermodel status in recent years by detailing how complex traits evolve in vertebrates. David Kingsley, evolutionary biologist at Stanford University, and colleagues generated a genome-wide linkage map by crossing two different species. The resulting data have detailed that a single gene, rather than small changes in many genes, can have a major impact on features such as the armor of these isolated lake fish—altering the course of evolution [7]. Using the map, they can now identify the genes controlling variable morphologies and behavioral ecology related to reproduction and mate choice. Given the success, the stickleback ensured that its genome would be sequenced, which will be completed later this year.

To really take a biological system down to the deepest mechanistic levels, Kingsley believes that researchers need all the types of methods that are routinely used in the most successful model organisms. “In the long run, the systems we are going to understand the best are the ones where you have not only arrays and RNAi, but also methods for crossing animals, mapping traits, cloning traits, doing sophisticated embryology, decreasing and increasing the function of particular pathways, and transferring specific genomic changes from one population into another,” he adds.

## Encore

All the world's a stage—especially for biologists. Until now, the few genetic superstar model systems delivered the bevy of biological information applicable to the cast of thousands. Scientists now have the tools to determine the roles played by some of the unique and interesting supporting characters.

Functional genomics has added a plot twist, as well as an element of suspense for ecological and evolutionary discovery. Mitchell-Olds foresees rapid results from functional genomics approaches. “In the next five to ten years, I think it will be feasible to identify genes controlling ecological important variation, and understand their functional effects in field, ecological consequences, and the historical and evolutionary forces that have influenced genetic variation for ecologically-important traits,” he says.

Can nonmodel species replace the genetic model species? It's doubtful [8]. “One should not underestimate the critical mass effect, which gives classical model systems a permanent advantage,” Chourrout notes, adding that forward genetic approaches used in model genetic organisms are a more efficient way to reveal unsuspected mechanisms.

Couple that with the wealth of knowledge and large research communities, and it's easy to see that the genetic organisms will continue in biology's starring roles. But the new cast of characters will be able to tell a richer story.

Additional ReadingCrawfordD2001Functional genomics does not have to be limited to a few select organismsGenome Biol21210.1186/gb-2001-2-1-interactions1001PMC15043511178276OleksiakMFRoachJLCrawfordDL2005Natural variation in cardiac metabolism and gene expression in Fundulus heteroclitus
Nat Genet3767721556802310.1038/ng1483PMC1447534SteinmetzLMDavisRW2004Maximizing the potential of function genomicsNat Rev Genet51902011497082110.1038/nrg1293WhiteKP2001Functional genomics and the study of development, variation and evolutionNat Rev Genet25285371143335910.1038/35080565

## Abbreviation

RNAi: RNA interference

## References

[pbio-0030219-Crawford1] Crawford D (2001). Functional genomics does not have to be limited to a few select organisms. Genome Biol.

[pbio-0030219-Oleksiak1] Oleksiak MF, Roach JL, Crawford DL (2005). Natural variation in cardiac metabolism and gene expression in Fundulus heteroclitus. Nat Genet.

[pbio-0030219-Steinmetz1] Steinmetz LM, Davis RW (2004). Maximizing the potential of function genomics. Nat Rev Genet.

[pbio-0030219-White1] White KP (2001). Functional genomics and the study of development, variation and evolution. Nat Rev Genet.

